# EBV status has prognostic implication among young patients with angioimmunoblastic T‐cell lymphoma

**DOI:** 10.1002/cam4.2742

**Published:** 2019-12-02

**Authors:** Ahmed E. Eladl, Kazuyuki Shimada, Yuka Suzuki, Taishi Takahara, Seiichi Kato, Kei Kohno, Ahmed Ali Elsayed, Chun‐Chieh Wu, Takashi Tokunaga, Tomohiro Kinoshita, Mamiko Sakata‐Yanagimoto, Shigeo Nakamura, Akira Satou

**Affiliations:** ^1^ Department of Pathology and Laboratory Medicine Nagoya University Hospital Nagoya Japan; ^2^ Department of Pathology Faculty of Medicine Mansoura University Mansoura Egypt; ^3^ Department of Hematology and Oncology Nagoya University Graduate School of Medicine Nagoya Japan; ^4^ Department of Surgical Pathology Aichi Medical University Hospital Nagakute Japan; ^5^ Department of Pathology and Molecular Diagnostics Aichi Cancer Center Hospital Nagoya Japan; ^6^ Department of Pathology Kaohsiung Medical University Hospital Kaohsiung Medical University Kaohsiung Taiwan; ^7^ Department of Hematology and Oncology Research Nagoya Medical Center Nagoya Japan; ^8^ Department of hematology and Cell Therapy Aichi Cancer Center Nagoya Japan; ^9^ Department of Hematology Faculty of Medicine University of Tsukuba Tsukuba Japan

**Keywords:** angioimmunoblastic T‐cell lymphoma, Epstein‐Barr virus, prognostic indicator, survival curve, young

## Abstract

Epstein‐Barr virus (EBV)‐positive B cells have been detected in 66%‐86% of patients with angioimmunoblastic T‐cell lymphoma (AITL). However, it remains controversial whether EBV status has an impact on the survival of patients with AITL. In this study, we aimed to reevaluate the impact of EBV on the clinicopathological characteristics of AITL. In particular, we focused on the impact of EBV in younger patients with AITL. In total, 270 cases of AITL were studied. Epstein‐Barr virus‐positive B cells were detected in 191 (71%) cases (EBER^+^ group). Among the patients who received anthracycline‐based therapy, the EBER status did not affect the overall survival (OS) or progression‐free survival (PFS). In the younger group of AITL (≤60 years), PFS was significantly worse in the EBER^−^ group compared to the EBER^+^ group (*P* = .0013). Furthermore, the multivariate analysis identified EBER‐negative status, thrombocytopenia, and elevated serum IgA level as significant adverse prognostic factors for PFS (*P* < .001, *P* < .001, and *P* = .002). Based on these findings, we constructed new prognostic model for the younger group, based on three adverse factors. We classified the patients into two risk groups: low risk (no or 1 adverse factor) and high risk (2 or 3 adverse factors). This new model for younger patients with AITL showed that both OS and PFS were significantly related to the level of risk (*P* < .0001). In summary, this study showed that, among younger patients with AITL, an EBER^+^ status significantly improved prognosis compared to an EBER^−^ status. Our new prognostic model should be applicable to younger patients with AITL.

## Graphical abstract

In the younger group of AITL, PFS was significantly worse in the EBER^−^ group compared to the EBER^+^ group (*P* = .0013). Furthermore, the multivariate analysis identified EBER‐negative status, thrombocytopenia, and elevated serum IgA level as significant adverse prognostic factors for PFS (*P* < .001, *P* < .001, and *P* = .002).

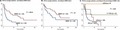

## 1 INTRODUCTION

T/Natural killer (T/NK)‐cell lymphomas represent 25% of all lymphoid neoplasms in Japan. Angioimmunoblastic T‐cell lymphoma (AITL) is the second most common entity among the T/NK‐cell lymphoma; it accounts for about 25%‐30% of T/NK‐cell lymphomas.^1^ AITL is an aggressive peripheral T‐cell lymphoma (PTCL) with distinctive pathological and clinical features.^2^, ^3^, ^4^ Gene expression studies have demonstrated that AITL is derived from follicular helper T cells (Tfh),^5^, ^6^ a distinctive subset of T‐helper cells that resides in lymphoid follicles and promotes the survival and differentiation of follicular B‐cells.^7^ The Tfh derivation of AITL explains many of its peculiar clinicopathological features, such as the admixture of B‐cells among neoplastic T‐cells, the association with follicular dendritic cell proliferations, and the presentation with hypergammaglobulinemia and autoimmune manifestations.^7^

Although the majority of AITL cases are observed among older individuals, AITL onset occurs within a wide range of ages. Patients mostly present with systemic disease in an advanced clinical stage, and it is frequently associated with an immune dysfunction and positive autoantibodies.^2^, ^3^, ^8^, ^9^ Abundant tumor‐infiltrating B cells are observed in AITL lesions. Previous studies have detected scattered Epstein‐Barr virus (EBV)‐positive B cells in 66%‐70% of Japanese patients with AITL.^9^, ^10^, ^11^ These tumor‐infiltrating B‐cells may show monoclonal immunoglobulin gene rearrangements, and they might subsequently progress to EBV‐positive (EBV^+^) or, rarely, EBV‐negative (EBV^−^) B‐cell lymphomas.^12^, ^13^, ^14^ The reactivation of EBV in AITL is thought to be an event secondary to an associated immune dysfunction. On the other hand, EBV^+^ B cells could have been detected very early in the disease course of AITL, which implies that it may also play a role in the development of AITL.^4^, ^12^, ^15^

It remains controversial whether EBV status has an impact on the survival of patients with AITL.^11^, ^16^, ^17^, ^18^ In this study, we aimed to reevaluate the impact of EBV on the clinicopathological characteristics of AITL. In particular, we focused on the impact of EBV in younger patients with AITL; this issue has not been well addressed to date.

## 2 MATERIALS AND METHODS

### 2.1 Patient sample

This retrospective study enrolled 270 patients who had been diagnosed with AITL between 1990 and 2016. Data for 108 cases were retrieved from the consultation files of Nagoya University database, and data for 162 cases were retrieved from a previous study,^9^ after updating with follow‐up data. Diagnoses were established based on histopathological and immunohistochemical criteria in accordance with the WHO classification.^19^ All cases were independently reviewed by three pathologists (authors AE, AS, and SN) to confirm the diagnosis and immunophenotype. Clinical, laboratory and follow‐up data were obtained from patient medical records at each institution. Involved sites were typically examined with a biopsy or radiographic evaluation (eg computed tomography or positron emission tomography). The Institutional Review Board of Nagoya University approved the study protocol.

### 2.2 Histological and immunohistochemical staining

Tissue samples were fixed in 10% formalin and embedded in paraffin. Tissue sections (5‐μm‐thick) were stained with hematoxylin and eosin. Immunoperoxidase studies were performed on formalin‐fixed paraffin‐embedded sections. The following monoclonal antibodies were used: CD3, CD8, CD10, CD30, BCL6 (DAKO), CD4, perforin (Novocastra Laboratories), PD1 (Abcam), CXCL13 (R&D systems), TIA‐1 (Coulter Immunology), granzymeB (Mososan), CD23 (Nichirei), and CD56 (eBioscience). Antibodies were applied after heating specimens in a microwave oven for antigen retrieval. Tissue samples were considered positive when more than 30% of the tumor cells were positive.

### 2.3 In situ hybridization study

The presence of EBV‐specific small RNAs was examined with in situ hybridization. Briefly, we applied EBV‐encoded small nuclear early region (EBER) oligonucleotides to formalin‐fixed, paraffin‐embedded sections as described previously.^20^

### 2.4 Detection of the *RHOA* G17V mutation

The *RHOA* G17V mutation was assessed with two methods: deep sequencing and a droplet digital PCR assay. DNA was extracted from formalin‐fixed paraffin embedded tissue with a silica membrane‐based DNA purification method (QIAamp DNA FFPE Tissue Kit, 56404; Qiagen KK). Deep sequencing and digital PCR assays were carried out as described previously.^21^, ^22^ We concluded the tumor had an *RHOA* G17V mutation, when the mutation was detected using both methods.

### 2.5 *T-cell receptor γ* (*TCRγ*) and *immunoglobulin H* (*IgH*) PCR study

DNA was extracted from formalin‐fixed tissue, and PCR analysis of the TCRγ and IgH genes carried out using the BIOMED2 protocol as described previously.^23^

### 2.6 Statistical analysis

Correlations between two groups were determined with Fisher's exact test, the Student's *t* test, and the Mann‐Whitney *U* test, as appropriate. Patient survival data were analyzed with the Kaplan‐Meier method. Differences in survival were tested with the log‐rank test. Overall survival (OS) was calculated, starting from the diagnosis date, and ending on the date of death or the date of the last follow‐up. Progression‐free survival (PFS) was calculated, starting from the diagnosis date, and ending on the first date of disease progression, relapse or death from any cause or the last date of follow‐up. Results that showed *P* < .05 were considered statistically significant.

## 3 RESULTS

### 3.1 EBER status of AITL

**Figure 1:**
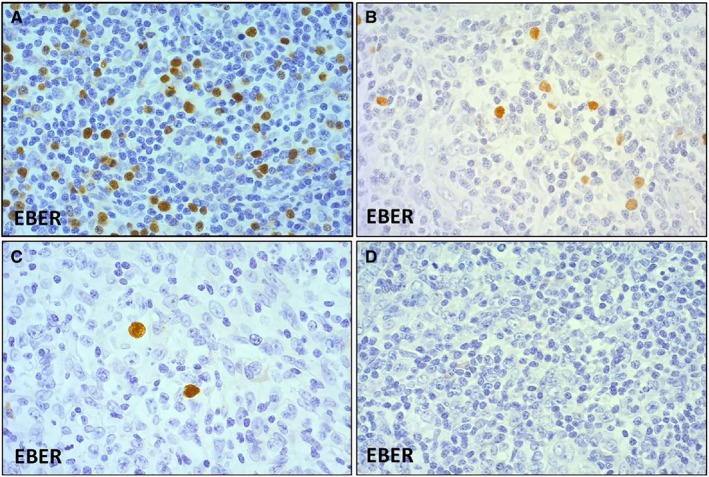
Younger patients (≤60 y) with angioimmunoblastic T‐cell lymphoma were classified into four groups based on the percentage of EBER^+^ cells among total tissue cellularity. A, ≥5% (EBER × 20), (B) 1~<5% (EBER × 20), (C) ≤1% (EBER × 20), and (D) 0% (EBER × 20). EBER, EBV‐encoded small nuclear early region

We first divided all patients with AITL into two groups, based on whether EBER^+^ B cells were detectable or undetectable in the background. Special attention was paid to the diagnoses of cases devoid of any EBER^+^ B‐cells. Scattered EBV‐infected B cells were detected in 191 cases (71%); these patients were considered the EBER‐positive (EBER^+^) group. The other 79 cases with no EBER^+^ B cells in the background were considered the EBER‐negative (EBER^‐^) group. Among patients with AITL that were ≤60 years old (n = 53), EBER^+^ B cells were detected in 30 cases (57%). We classified these 53 patients into four groups based on the percentage of EBER^+^ cells in the total tissue cellular population; the groups were ≥5%, 1‐<5%, ≤1%, and 0%. Representative micrographs of each group are shown in Figure 1.

As mentioned above, EBER^+^ B cells were not detected in 79 cases (29%). Some may assert that these cases are not likely to be diagnosed as AITL, because EBER^+^ B cells are present in majority of AITL cases, and it is one of main pathological findings of AITL. Particularly, these cases should be thoroughly differentiated from nodal PTCL with Tfh phenotype. We carefully reevaluated the cases without EBER^+^ B cells and confirmed the diagnosis of AITL based on the pathological findings as follow. The pathological findings include intermediate to medium‐sized neoplastic T cells with clear to pale cytoplasm and proliferation of high endothelial venules and follicular dendritic cells.

### 3.2 Clinical characteristics and survival of patients, relative to EBER status

Table 1 summarizes the clinical characteristics of 270 patients with AITL. The cohort included 167 males and 103 females, with a median age of 70 years (range, 32‐91 years). Of these, 235 patients (87%) were categorized as stage III/IV AITL; 171 patients (67%) were classified as high‐intermediate or high risk, according to the International Prognostic index (IPI); and 182 patients (71%) were classified as groups 3 and 4, according to the Prognostic index of T‐cell lymphoma (PIT).

**Table 1:** Clinicopathological features of 270 AITL patients

Variable	Total patients (n = 270)	EBER‐positive (n = 191)	EBER‐negative (n = 79)	*P* value
Age, median (range)	70 (32‐91)	71 (32‐88)	68 (38‐91)	.097
Age > 60 y	217/270 (80%)	161/191 (84%)	56/79 (71%)	.011
Sex, male	167/270 (62%)	115/191 (60%)	52/79 (66%)	.235
Extranodal > 1	60/267 (23%)	42/190 (22%)	18/77 (23%)	.470
Stage III/IV	235/270 (87%)	166/191 (87%)	69/79 (87%)	.549
B‐Symptoms	141/260 (54%)	100/183 (55%)	41/77 (53%)	.471
PS > 1	89/259 (34%)	65/184 (35%)	24/75 (32%)	.359
IPI HI/H	171/257 (67%)	124/183 (68%)	47/74 (634%)	.304
PIT groups 3/4	182/258 (71%)	131/183 (72%)	51/75 (68%)	.334
WBC > 10 000/mm^3^	66/269 (25%)	47/190 (25%)	19/79 (24%)	.519
Hb < 10.5 g/dL	67/269 (25%)	49/190 (26%)	18/79 (23%)	.361
Plate < 150 000/mm^3^	85/259 (33%)	61/185 (33%)	24/74 (32%)	.528
Alb < 3.5 g/dL	136/258 (53%)	102/182 (56%)	34/76 (45%)	.064
LDH > normal	184/268 (69%)	130/189 (69%)	54/79 (68%)	.527
sIL‐2R > 4000 U/mL	139/251 (55%)	101/179 (56%)	38/72 (53%)	.349
CRP > 2.00 mg/dL	102/252 (41%)	74/182 (41%)	28/70 (40%)	.521
IgG > 1700 mg/dL	109/215 (51%)	83/151 (55%)	26/64 (41%)	.038
IgM > 200 mg/dL	92/212 (43%)	66/150 (44%)	26/62 (42%)	.452
IgA > 400 mg/dL	76/212 (36%)	59/150 (39%)	17/62 (27%)	.067
CR rate^a^	114/197 (58%)	71/132 (54%)	43/65 (66%)	.066
Relapse/progression	179/270 (66%)	118/191 (62%)	61/79 (77%)	.010

Abbreviations: AITL, angioimmunoblastic T‐cell lymphoma; Alb, albumin; CR, complete remission; CRP, c‐reactive protein; H, high; HI, high‐intermediate; Hb, hemoglobin; IPI, international prognostic index; LDH, lactate dehydrogenase; PS, performance status; PIT, prognostic index of T‐cell lymphoma; sIL‐2R, soluble interleukin‐2 receptor; WBC, white blood cell.

a CR rate of patients who received anthracycline‐containing combination chemotherapy.

Compared to the EBER^−^ group, EBER^+^ group had a significantly higher frequency of patients older than 60 years (*P* = .011). EBER^+^ AITL group tended to show a higher age distribution (median, 71 vs 68 years; *P* = .097). Laboratory analyses showed that, at presentation, the EBER^+^ group showed higher elevation of serum IgG levels (*P* = .038) and tended to show higher IgA levels (*P* = .067) than the EBER^−^ group. Among the patients who received anthracycline‐based therapy, the EBER status did not affect the OS or PFS (*P* = .90 and .16, respectively; Figure S1).

### 3.3 Clinicopathological features of patients with AITL, according to age and EBER status

**Figure 2:**
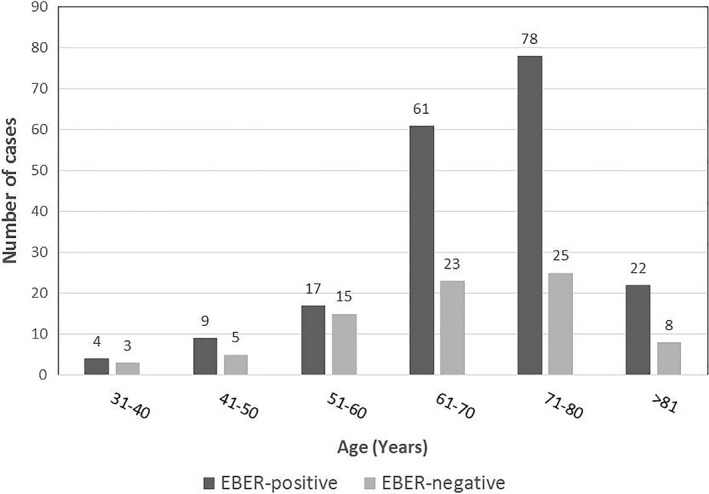
EBER positive and negative status across the different age groups of patients with angioimmunoblastic T‐cell lymphoma. The frequency of EBER‐postive cases surged in patients older than 60 y. EBER, EBV‐encoded small nuclear early region

To investigate whether the impact of EBER expression in patients with AITL depended on age, we divided all patients with AITL into two groups based on age; ages ≤ 60 (younger group) and ages >60 years (older group). We selected 60 years as the cut‐off age because, in our cohort, the frequency of EBER expression surged in patients older than 60 years (Figure 2). Then, we compared the clinicopathological features between patients with EBER^+^ and EBER^−^ patients in both age groups (Table 2).

**Table 2:** Clinical characteristics according to EBER status in different age groups

Variable	AITL patients ≤ 60 y (n = 53)			AITL patients > 60 y (n = 217)		
	EBER‐positive (n = 30)	EBER‐negative (n = 23)	*P*	EBER‐positive (n = 161)	EBER‐negative (n = 56)	*P*
Age, median	51.5	54	.276	73	73.5	.971
Sex, male	24/30 (80%)	18/23 (78%)	.570	91/161 (57%)	34/56 (61%)	.350
Extranodal > 1	10/30 (33%)	8/23 (35%)	.570	32/160 (20%)	10/54 (19%)	.493
Stage III/IV	26/30 (87%)	19/23 (83%)	.486	140/161 (87%)	50/56 (89%)	.424
B symptoms	15/28 (54%)	12/22 (55%)	.586	85/155 (55%)	29/55 (53%)	.454
PS > 1	8/30 (27%)	5/22 (23%)	.503	57/154 (37%)	19/53 (36%)	.508
IPI HI/H	11/30 (37%)	6/22 (27%)	.341	113/153 (74%)	41/52 (79%)	.301
PIT groups 3/4	11/30 (37%)	8/22 (36%)	.607	120/153 (78%)	43/53 (81%)	.420
WBC > 10 000/mm^3^	7/30 (23%)	6/23 (26%)	.533	40/160 (25%)	13/56 (23%)	.471
Hb < 10.5 g/dL	5/30 (17%)	5/23 (22%)	.451	44/160 (28%)	13/56 (23%)	.330
Plate < 150 000/mm^3^	7/29 (24%)	8/22 (36%)	.261	54/156 (35%)	16/52 (31%)	.371
Alb < 3.5 g/dL	10/28 (36%)	9/23 (39%)	.515	92/154 (59%)	25/53 (47%)	.076
LDH > normal	18/30 (60%)	14/23 (61%)	.588	112/159 (70%)	40/56 (71%)	.517
sIL‐2R > 4000 U/mL	16/29 (55%)	11/21 (52%)	.536	85/150 (57%)	27/51 (53%)	.381
CRP > 2.00 mg/dL	10/28 (36%)	3/21 (14%)	.086	64/154 (42%)	25/49 (51%)	.159
IgG > 1700 mg/dL	10/22 (46%)	9/18 (50%)	.512	73/129 (57%)	17/46 (37%)	.017
IgM > 200 mg/dL	10/22 (46%)	11/18 (61%)	.252	56/128 (44%)	15/44 (34%)	.172
IgA > 400 mg/dL	6/22 (27%)	5/18 (28%)	.623	53/128 (41%)	12/44 (27%)	.067
CR rate^a^	12/22 (55%)	16/20 (80%)	.077	59/110 (54%)	27/45 (60%)	.293
Relapse/progression	17/30 (57%)	20/23 (87%)	.017	101/161 (62%)	41/56 (73%)	.103

Abbreviations: AITL, angioimmunoblastic T‐cell lymphoma; Alb, albumin; CR, complete remission; CRP, c‐reactive protein; H, high; HI, high‐intermediate; Hb, hemoglobin; IPI, international prognostic index; LDH, lactate dehydrogenase; PS, performance status; PIT, prognostic index of T‐cell lymphoma; sIL‐2R, soluble interleukin‐2 receptor; WBC, white blood cell.

a CR rate of patients who received anthracycline‐containing combination chemotherapy.

In the younger group (n = 53), 45 patients (85%) were treated with anthracycline‐containing combination chemotherapy, as follows: 33 patients received the combination of cyclophosphamide, vincristine, adriamycin, and prednisolone (CHOP); 6 patients received the combination of pirarubicin, cyclophosphamide, vincristine, and prednisolone (THP‐COP); and 6 patients received second/third‐generation chemotherapy. Autologous stem cell bone marrow transplantations were performed in 21 patients. Complete remission (CR) was achieved in 28/45 (62%) patients who received anthracycline‐based therapies. The EBER^+^ group tended to achieve CR less frequently than the EBER^−^ group, although the difference was not significant (*P* = .077). However, the EBER^+^ group showed a significantly lower frequency of relapse/progression compared to the EBER^−^ group (*P* = .017).

On the other hand, EBER expression was more common in the older group (n = 217). Among these patients, EBER^+^ B cells were detected in 161 cases (74%). Moreover, in this older group, EBER expression was associated with elevated serum immunoglobulin G (IgG) (*P* = .017), elevated serum immunoglobulin A (IgA) (*P* = .067), and low serum albumin (*P* = .076) levels. Among these patients, 160 received anthracycline‐containing combination chemotherapy, as follows: CHOP (n = 96), THP‐COP (n = 57), and second/third‐generation chemotherapy (n = 7). Four patients received autologous stem cell bone marrow transplantations. Eighty‐six (55%) patients achieved CR, with no significant difference between the EBER^+^ and EBER^−^ groups.

### 3.4 Prognosis of each age group according to EBER status

**Figure 3:**
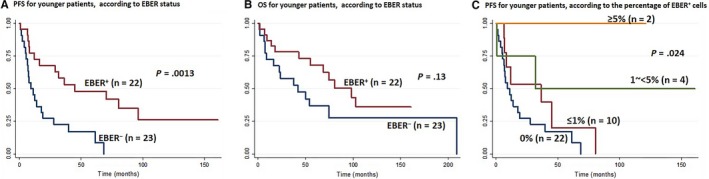
Kaplan‐Meier survival curves for younger patients (≤60 y) with angioimmunoblastic T‐cell lymphoma, according to EBER status. A, Progression‐free survival (PFS), according to EBER status; (B) overall survival, according to EBER status; (C) PFS, according to the percentage of EBER^+^ cells. EBER, EBV‐encoded small nuclear early region

Among the patients in the younger group who received anthracycline‐based therapy, PFS was significantly worse in the EBER^−^ group compared to the EBER^+^ group (*P* = .0013; Figure 3A). In addition, OS was worse in the EBER^−^ group than in EBER^+^ group, although the difference was not significant (*P* = .13; Figure 3B). Notably, we found that the percentage of EBER^+^ cells in this younger group had a significant impact on PFS. A low percentage of EBER^+^ cells was significantly correlated with a worse PFS (*P* = .024; Figure 3C), but it had no impact on the OS (data not shown).

On the other hand, in older patients with AITL, the EBER status did not affect the PFS or OS (*P* = .99 and *P* = .75, respectively).

### 3.5 Risk factors for OS and PFS in patients with AITL ≤ 60 years old

A univariate analysis revealed four adverse prognostic factors for OS in younger patients, including: male sex (*P* = .043), thrombocytopenia (*P* = .010), elevated serum sIL‐2R levels (*P* = .050), and elevated serum IgA levels (*P* = .002). The multivariate analysis identified both thrombocytopenia and elevated serum IgA levels as significant prognostic factors for OS (*P* = .001 and .004, respectively; Table 3).

**Table 3:** Risk factors for overall survival (patients ≤ 60 y)

Variable	Univariate analysis			Multivariate analysis		
	HR	95% CI	*P*	HR	95% CI	*P*
Sex, male	4.46	1.04‐18.97	.043	2.76	0.52‐14.56	.230
Extranodal > 1	1.21	0.52‐2.81	.649			
Stage III/IV	1.24	0.42‐3.63	.683			
IPI HI/H	1.40	0.59‐3.27	.436			
PIT group ¾	1.78	0.79‐3.99	.161			
B symptoms	1.56	0.69‐3.55	.280			
PS > 1	1.95	0.79‐4.82	.147			
WBC > 10 000/mm^3^	0.92	0.37‐2.31	.873			
Hb < 10.5 g/dL	1.49	0.59‐3.72	.392			
Plate < 150 000/mm^3^	3.03	1.30‐7.05	.010	11.17	2.73‐45.72	.001
Alb < 3.5 g/dL	1.62	0.73‐3.58	.232			
LDH > normal	2.10	0.93‐4.74	.073			
sIL‐2R > 4000 U/mL	2.48	0.99‐6.17	.050	2.71	0.79‐9.26	.110
IgG > 1700 mg/dL	0.97	0.38‐2.49	.964			
IgM > 200 mg/dL	1.44	0.55‐3.72	.451			
IgA > 400 mg/dL	4.88	1.80‐13.23	.002	7.52	1.90‐29.74	.004
EBER‐negative	1.80	0.82‐3.94	.137			

Abbreviations: Alb, albumin; CR, complete remission; CRP, C‐reactive protein; H, high; HI, high‐intermediate; Hb, hemoglobin; HR, hazard ratio; IPI, international prognostic index; LDH, lactate dehydrogenase; PS, performance status; PIT, prognostic index of T‐cell lymphoma; sIL‐2R, soluble interleukin‐2 receptor; WBC, white blood cell.

The univariate analysis also identified thrombocytopenia (*P* = .002), EBER‐negative status (*P* = .002), and elevated serum IgA levels (*P* = .051) as three adverse prognostic factors for PFS. Furthermore, the multivariate analysis identified the same three prognostic factors for PFS (*P* < .0001, *P* < .0001, and *P* = .002, respectively; Table 4).

**Table 4:** Risk factors for progression‐free survival (patients ≤ 60 y)

Variable	Univariate analysis			Multivariate analysis		
	HR	95% CI	*P*	HR	95% CI	*P*
Sex, Male	1.27	0.52‐3.09	.597			
Extranodal > 1	0.83	0.38‐1.78	.638			
Stage III/IV	0.83	0.36‐1.94	.682			
IPI HI/H	0.77	0.35‐1.73	.543			
PIT group 3/4	1.31	0.63‐2.75	.463			
B symptoms	1.45	0.72‐2.92	.288			
PS > 1	1.19	0.51‐2.77	.681			
WBC > 10 000/mm^3^	1.09	0.49‐2.42	.830			
Hb < 10.5 g/dL	1.25	0.54‐2.88	.600			
Plate < 150 000/mm^3^	3.34	1.53‐7.25	.002	8.07	2.83‐23.00	<.001
Alb < 3.5 g/dL	1.46	0.72‐2.93	.286			
LDH > normal	1.13	0.57‐2.25	.707			
sIL‐2R > 4000 U/mL	1.21	0.59‐2.49	.600			
IgG > 1700 mg/dL	1.39	0.63‐3.05	.407			
IgM > 200 mg/dL	1.25	0.572‐2.74	.573			
IgA > 400 mg/dL	2.22	0.99‐4.97	.051	4.12	1.66‐10.19	.002
EBER‐negative	3.25	1.52‐6.92	.002	5.82	2.18‐15.55	<.001

Abbreviations: Alb, albumin; CR, complete remission; CRP, C‐reactive protein; H, high; HI, high‐intermediate; Hb, hemoglobin; HR, hazard ratio; IPI, international prognostic index; LDH, lactate dehydrogenase; PS, performance status; PIT, prognostic index of T‐cell lymphoma; sIL‐2R, soluble interleukin‐2 receptor; WBC, white blood cell.

**Figure 4:**
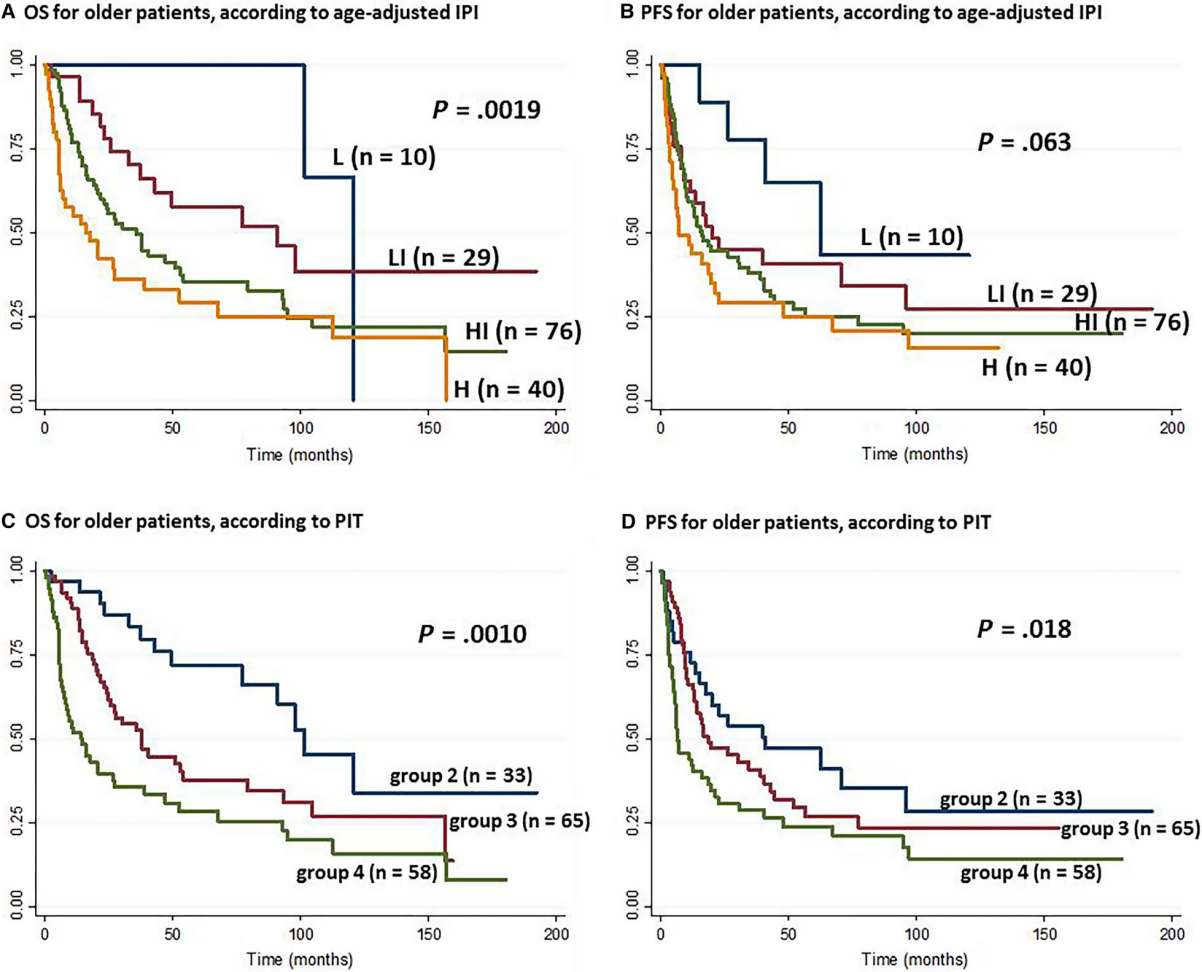
Kaplan‐Meier survival curves, according to age‐adjusted international prognostic index (IPI) and prognostic index of T‐cell lymphoma (PIT) in older patients with angioimmunoblastic T‐cell lymphoma (>60 y). A, Overall survival (OS) and (B) progression‐free survival (PFS), according to age‐adjusted IPI; the patients were classfied into low (L) low‐intermediate (LI), high‐intermediate (HI), and high (H). C, OS and (D) PFS, according to PIT; the patients were classified into group 1‐4. None of the patients was classified as group 1

**Figure 5:**
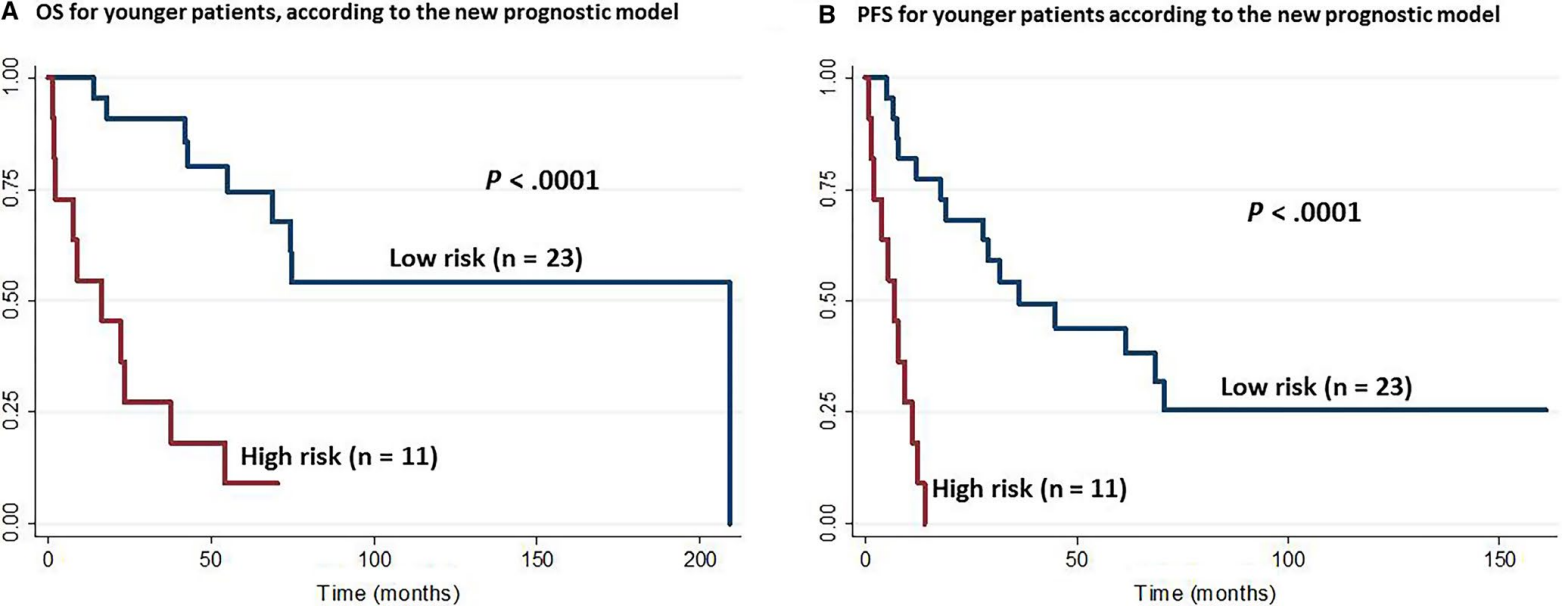
Kaplan‐Meier survival curves for younger patients (≤60 y) with angioimmunoblastic T‐cell lymphoma, according to the new prognostic model. A, Overall survival (OS) and (B) progression‐free survival (PFS), according to the new prognostic model; the patients were classified into two risk groups: low risk (no or 1 adverse factor) and high risk (2 or 3 adverse factors)

Both the OS and the PFS were influenced by the age‐adjusted IPI and the PIT in the older group (Figure 4) but not in the younger group (Figure S2). Therefore, we constructed another prognostic model for the younger group, based on three adverse factors: thrombocytopenia, EBER‐negative status and elevated serum IgA levels. We classified the patients into two risk groups: low risk (no or 1 adverse factor) and high risk (2 or 3 adverse factors). This new model for younger patients with AITL showed the both OS and PFS were significantly related to the level of risk (*P* < .0001; Figure 5). The 1‐ and 3‐year OS rates were 95 and 91%, respectively, in the low risk group (n = 23 patients, 68%). The 1‐ and 3‐year OS rates were 45% and 18%, respectively, in the high risk group (n = 11 patients, 32%). The 1‐ and 3‐year PFS rates were 77% and 49%, respectively, in the low risk group, and 9% and 0%, respectively, in the high risk group.

### 3.6 PCR results

PCR studies for TCRγ and IgH gene rearrangement were performed in 33 cases with available material of younger AITL patients (≤60 years). Clonal TCRγ gene rearrangement was detected in 6 cases and clonal IgH gene rearrangement in one case. The detection rate of TCRγ gene rearrangement was not significantly different between EBER^+^ (3/19 patients; 16%) and EBER^−^ (3/14 patients; 21%) groups. One case with clonal IgH gene rearrangement was the EBER^+^ case.

### 3.7 *RHOA* mutations

*RHOA* mutation analysis was performed in 23 younger patients (≤60 years) with AITL that had material available for analysis. *RHOA* mutations were detected in 15 cases (65%). The mutation incidence was not significantly different between the EBER^+^ (8/13 patients; 62%) and EBER^−^ (7/10 patients; 70%) groups.

## 4 DISCUSSION

This study included 270 patients with AITL. Although 162 of those patients were included in the previous report,^9^ the impact of EBER positivity on prognosis within different age groups had not been investigated. In this study, AITL manifested as a disease that mostly affected older individuals, with an aggressive behavior and frequent relapses. These clinical features were consistent with previous reports.^2^, ^3^, ^8^ Epstein‐Barr virus‐encoded small nuclear early region expression was detected in 71% of our patients comparable to rates reported in several previous studies.^3^, ^8^, ^11^ Previous studies could not resolve the controversy over whether EBV status impacted survival in patients with AITL.^11^, ^16^, ^17^, ^18^

In this study, we investigated the impact of EBER status on patients with AITL within different age groups. In our cohort, 80% of patients were older than 60 years, and the incidence of EBER positivity was significantly higher in this age group compared to the younger group (74% vs 57%; *P* = .011). Furthermore, we revealed that, among all patients with AITL and among older patients with AITL, EBER^+^ was associated with elevated serum IgG and IgA levels and low serum albumin levels. However, EBER expression showed no significant impact on survival in older patients. This peculiar association between AITL, EBER status, and elevated serum immunoglobulin levels might be explained by the Tfh‐like behavior of the neoplastic T‐cells in AITL. We speculated that, like Tfh‐like cells, AITL tumor cells suppressed the conventional CD4^+^ T cells, via IL10 and transformin growth factor β (TGF‐β). This suppression could have resulted in defective T‐cell responses and the reactivation of EBV in the clinical course of patients with AITL. Therefore, in patients with AITL, an EBER^+^ status might imply tumor cells with a more pronounced Tfh‐like nature compared to tumor cells in patients with an EBER^−^ status. Tfh cells are also responsible for B‐cell differentiation and antibody production, in response to certain cytokines (IL21 and IL10). This role might be also enhanced in EBER^+^ AITL, which might then lead to elevated serum IgG and IgA levels.^7^, ^24^, ^25^, ^26^

On the other hand, among younger patients with AITL, EBER expression did not affect the clinicopathological parameters significantly, except for prognosis. We found that EBER expression was associated with a higher PFS (*P* = .0013) and the percentage of EBER^+^ cells had an impact on the PFS. A worse PFS was significantly correlated with a low percentage of EBER^+^ cells (*P* = .024). Although patients with EBER^+^ status were characterized by a tendency to achieve CR less frequently (*P* = .077), they exhibited a significantly lower frequency of relapse/progression (*P* = .017), compared to patients with EBER^−^ status. These results suggested that among younger patients, an EBER^+^ status might be associated with an indolent clinical course and a better prognosis compared to an EBER^−^ status. The low sensitivity to chemotherapy might be due to an indolent clinical disease behavior in this group. Indeed, it is well known that indolent lymphomas are less sensitive to chemotherapy than aggressive lymphomas. Taken together, our findings suggested that younger patients with EBER^+^ status formed a distinct group among patients with AITL.

In our cohort, we assessed the clonal rearrangements of *TCR*γ and *IgH* of younger patients with AITL. As mentioned, clonal TCRγ and IgH gene rearrangements were detected in 18% (6/33) and 3% (1/33) of tested cases, respectively. According to previous reports, clonal rearrangements of *TCR*γ and *IgH* were detected in 75%‐90% or 25%‐30% of cases with AITL.^19^ Therefore, based on our finding, younger patients with AITL had a lower rate of clonal rearrangements of *TCR*γ and *IgH*. This finding evoked in us the historical issue of AITL, if this disease should be regarded as either of lymphoma or atypical reactive lesion. So far examined here, we could not find any difference in their histopathological features between younger and older groups. We believe that this study sheds light on this historical issue once again, which appeared to be underestimated in the last two decades. Future studies are expected in this field.

We hypothesized that the specific features of younger patients with AITL and EBER^+^ could be due to a different pathogenesis. To test this hypothesis, we assessed *RHOA* mutations in younger patients with AITL *RHOA* mutations were detected in 65% of the younger AITL group, with no significant difference between EBER^+^ (62%) and EBV^−^ (70%) status. In previous reports, *RHOA* mutations were detected in 50%‐70% of patients with AITL consistent with tour findings.^27^, ^28^, ^29^ Sakata‐Yanagimoto et al recently reported that all cases with the *RHOA* G17V mutation also had *TET2* mutations.^27^ However, *TET2* and *DNMT3A* mutations were found in nontumor cells of patients with AITL and even in blood cells of healthy individuals.^30^, ^31^ On the other hand, *RHOA* and *IDH2* mutations were detected only in tumor cells. Based on these findings, a multistep tumorigenesis model was proposed for AITL.^32^ In that model, *TET2* and *DNMT3A* mutations occurred as initial events, and *RHOA* and *IDH2* mutations were acquired later in AITL development. Considering the frequency of *RHOA* mutations in young patients with AITL and EBER^+^ status, we suggest that the multistep tumorigenesis model might be applicable to these patients. Further investigation is required to reveal the mechanism underlying the specific features of younger patients with AITL and EBER^+^ status.

We found that thrombocytopenia and elevated serum IgA levels were significant prognostic factors for poor OS and PFS in younger patients (≤60 years) with AITL. The EBER^−^ status in this group was an additional prognostic factor for poor PFS. Thrombocytopenia (<150 × 10^9^/mm^3^) was detected in about 33% of our cases, irrespective of age. Previous reports documented thrombocytopenia in 20%‐40% of patients with AITL; moreover, in a fraction of those patients, thrombocytopenia was related to immune thrombocytopenic purpura.^33^, ^34^, ^35^ Previous studies also reported an association between thrombocytopenia and poor OS^33^, ^36^ and PFS.^33^

Among our patients, 36% presented with elevated serum IgA levels (>400 mg/dL), which tended to be associated with EBER positivity (*P* = .067). The Tfh‐like nature of AITL could be responsible for the elevation of IgA serum levels; Tfh cells produce TGF‐β1 and IL‐12, which bring about the induction and differentiation of IgA‐plasmablasts.^26^ A number of previous studies reported an association between AITL and IgA; in those studies, AITL was associated with atypical linear IgA dermatosis,^37^ IgA nephropathy,^38^ and leukocytoclastic vasculitis with IgA deposits.^39^ Kato et al reported that, in a series of Japanese patients with HTLV‐1‐negative nodal PTCL, elevated serum IgA levels were found in 47% of patients with AITL. They identified elevated serum IgA as a prognostic factor of poor OS, regardless of the histological PTCL subtype.^40^

It has been controversial whether IPI and PIT were significant factors in the prognosis of PTCL (including AITL).^3^, ^8^, ^41^, ^42^, ^43^, ^44^ In our study, both the age‐adjusted‐IPI and the PIT showed significant influences on the prognosis of AITL in older, but not in younger patients. On the other hand, our new prognostic model was significantly predictive of the prognosis in younger patients with AITL. Future studies are necessary to validate our findings.

In summary, this study showed that, among younger patients with AITL, an EBER^+^ status significantly improved prognosis compared to an EBER^−^ status. In addition, thrombocytopenia and elevated serum IgA levels were significant prognostic factors for poor OS and PFS among younger patients with AITL. Our new prognostic model, based on these three adverse prognostic factors, should be applicable to younger patients with AITL.

## CONFLICT OF INTEREST

None declared.
