# Supplementary figures and images for: EBV status has prognostic implication among young patients with angioimmunoblastic T‐cell lymphoma
Source: Cancer Med. 2019 Dec 2;9(2):678–88. doi: 10.1002/cam4.2742 (PMC6970042; doi:10.1002/cam4.2742)

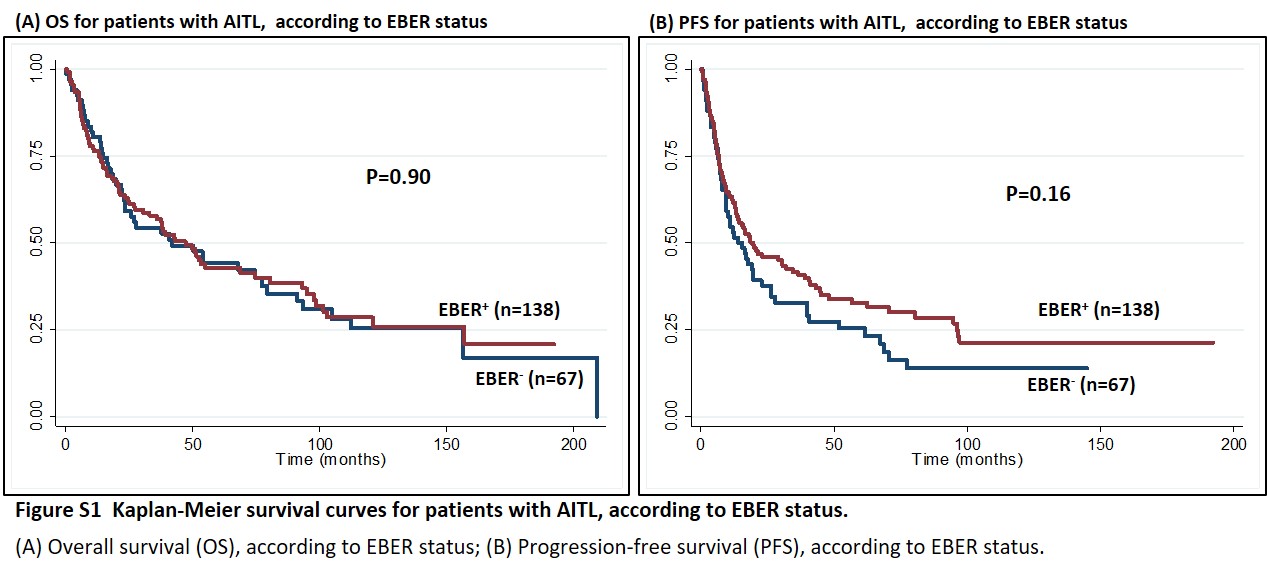

Supplement: Supplementary file 1 [file CAM4-9-678-s001.jpg]

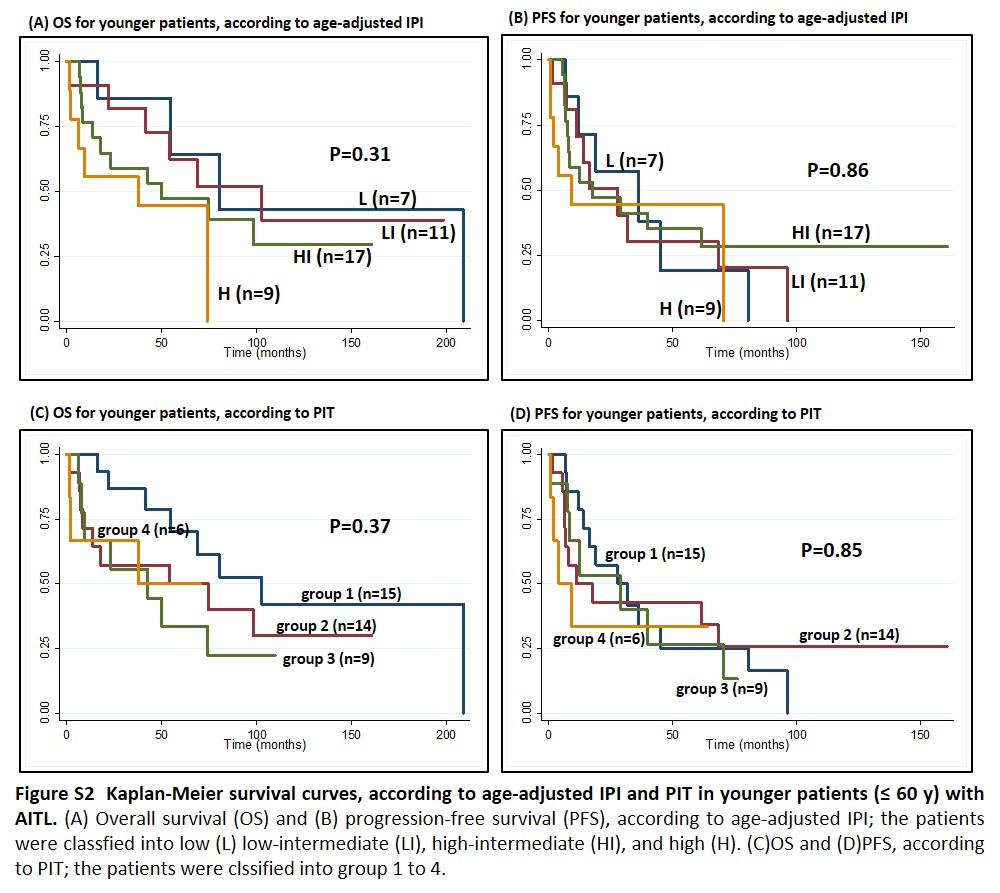

Supplement: Supplementary file 2 [file CAM4-9-678-s002.jpg]
