# Supplementary material for: EBV status has prognostic implication among young patients with angioimmunoblastic T‐cell lymphoma
Source: Cancer Med. 2019 Dec 2;9(2):678–88. doi: 10.1002/cam4.2742 (PMC6970042; doi:10.1002/cam4.2742)
Supplement: Supplementary file 3 [file CAM4-9-678-s003.docx]

**Supporting Information**

**Figure S1 Kaplan-Meier survival curves for patients with AITL, according to EBER status.**

**Figure S2 Kaplan-Meier survival curves, according to age-adjusted IPI and PIT in younger patients (≤ 60 y) with AITL.**
